# Surgical Treatment of a Patient with Human Tail and Multiple Abnormalities of the Spinal Cord and Column

**DOI:** 10.4061/2011/153797

**Published:** 2010-10-18

**Authors:** Chunquan Cai, Ouyan Shi, Changhong Shen

**Affiliations:** ^1^Department of Pediatric Neurosurgery, General Hospital of Tianjin Medical University, no. 154, Anshan Road, Heping District, Tianjin 300052, China; ^2^Department of Neurosurgery, Tianjin Children's Hospital, Tianjin 300074, China; ^3^Faculty of Basic Medicine, Tianjin Medical University, Tianjin 300070, China

## Abstract

The dorsal cutaneous appendage, or so-called human tail, is often considered to be a cutaneous marker of underlying occult dysraphism. The authors present a case of human tail occurring in a 9-month-old infant with multiple abnormalities of the spinal cord and spine. Examination revealed unremarkable except for a caudal appendage and a dark pigmentation area in the low back. Neuroradiological scans revealed cleft vertebrae and bifid ribbon, split cord malformations, block vertebrae, and hemivertebra. Surgical excision of the tail and untethering the spinal cord by removal of the septum were performed. The infant had an uneventful postoperative period and was unchanged neurologically for 18 months of followup. To our knowledge, no similar case reports exist in the literature. The specific features in a rare case with a human tail treated surgically are discussed in light of the available literature.

## 1. Introduction

A human tail is a rare congenital anomaly with a prominent lesion from the lumbosacrococcygeal region. Many authors saw this curious and rare condition to be evidence of man's descent from or relation to other animals, while others made it the subject of superstition. Advanced imaging technology in recent decades has allowed a more thorough investigation of these patients and better defined their association with spinal dysraphism and tethered spinal cord [1]. In this case report, we describe an infant who had combined anomalies, consisting of a human tail and multiple abnormalities of the spinal cord and spine. We also reviewed the documents of the patient with reference to literatures. 

## 2. Case Report

**Figure 1:**
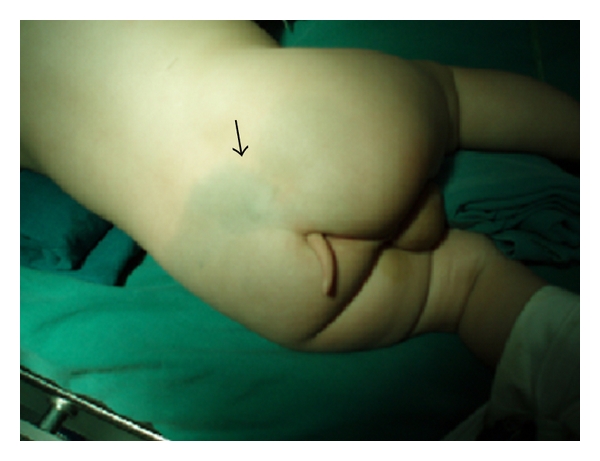
Photography of the infant showing the appendage was attached to the back tip of coccyx appearing like a human tail and a dark pigmentation area in the low back.

**Figure 2:**
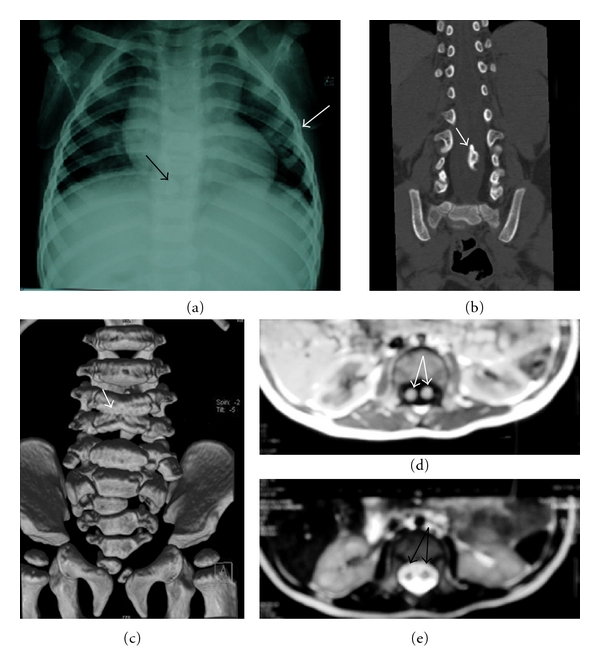
Plain radiographs revealed cleft vertebrae of the ninth thoracic vertebrae, and bifid rib of the fourth rib (Figure 2(a)). Computed tomography (CT) scans showed split cord malformations, block vertebrae of the third and fourth vertebrae, and hemivertebra of the second sacral vertebrae (Figures 2(b) and 2(c)). Magnetic resonance imaging (MRI) showed split cord malformations (Figure 2(d) and 2(e)).

**Figure 3:**
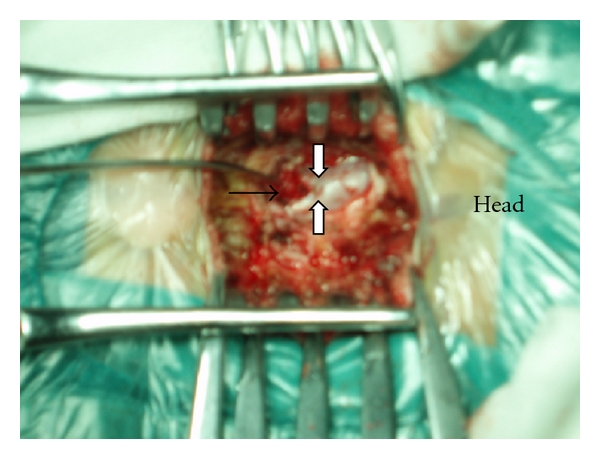
Photography during the operation showing two hemicords within two separate dural sleeves.

A 9-month-old healthy male infant was brought for consultation for a “tail like” structure in the lumbosacral area since birth. On physical examination, the appendage was 5 cm long and was attached to the back tip of coccyx appearing like a human tail (Figure 1). A dark pigmentation area in the low back was also found (Figure 1). According to the parents, the tail was about 2 cm at birth and the size had been increasing with age. It was soft and nontender, covered with normal skin. No bony attachment or any voluntary movement was observed in the mass. His muscle strength, tone, and sensation were intact with normal reflexes present. There was normal rectal tone with an anal wink reflex present bilaterally. The infant was born after an uneventful pregnancy. There was no history of any illness, exposure to radiation, or taking any drug during pregnancy. He did not have any family history of congenital abnormality. Plain radiographs revealed cleft vertebrae of the ninth thoracic vertebrae, and bifid rib of the fourth rib (Figure 2(a)). Computed tomography (CT) scans showed split cord malformations, block vertebrae of the third and fourth vertebrae, and hemivertebra of the second sacral vertebrae (Figures 2(b) and 2(c)). Magnetic resonance imaging (MRI) showed split cord malformations (Figure 2(d) and 2(e)). With the impression of human tail coexisting with type I split cord malformations, operation of resection of the tail, removing of the septum and spinal cord untethered, was done with continuous intraoperative monitoring of sensory evoked potentials and electromyogram recording of lower extremity and sphincter muscles. The tail was elliptically excised, and the subcutaneous portion sharply dissected from the dorsal lumbosacral fascia. Despite careful inspection for a fascial defect and subfascial extension of the tail structure, none was observed, and the tail was removed enbloc. The lumbar fascia was opened and L_4_–L_5_ laminoplasty performed. A bony septum originating from segmented lumbar vertebrae was found to divide the spinal cord. Two hemicords were shown to course within two separate dural sleeves (Figure 3). The spinal cord was untethered and decompressed by removal of the septum. The filum terminale was thickened and was transected. The other abnormalities of the spine were untreated. The infant was recovered uneventfully in the postoperative period. In the followup till 18 months, he was all right without any neurological deficit. His bowel and bladder habits were also normal.

## 3. Discussion

The dorsal cutaneous appendage, or so-called human tail, is considered to be a marker of underlying intraspinal pathology of occult spinal dysraphism [2]. However, certain authors have considered these to be a benign stigma without any cord malformations [3]. There have been many previous reports to date that spinal dysraphism is usually accompanied by several anomalies, including skin protrusion, pigmentation, sinus formation, human tail, and subcutaneous or spinal lipomas [4]. As a consequence, a multitude of spinal cord and spine anomalies associations including spina bifida, meningocele, lipomeningoceles, myelomeningocele, intraspinal lipoma, spinal cord tethering, coccygeal vertebrae have been described in patients with human tail [1, 5–9]. But no report similar to our patient exists in the literature. The present case demonstrates a cutaneous marker in the form of a tail at the back tip of coccyx coexisting with split cord malformations.

Dao and Netsky [10] reviewed 32 previous descriptions of tails published from 1859 to 1982. They distinguished true or persistent vestigial tails from other forms of caudal appendages or pseudotails. A true human tail is defined as a boneless, midline protrusion capable of spontaneousor reflex motion. The true human tail lacks vertebrae in all cases and is usually attached to the skin of the sacrococcygeal region. A pseudotail is a caudal protrusion composed of other normal and abnormal tissue. However, their classification is mainly based on histolopathological findings and is not done from an embryological standpoint. The associated embryogenesis of the human tail is first noted at the fourth week of gestation. The somites are formed, and the remaining primitive knot and streak compose a compact mass at the caudal end of the embryo that is called the tail bud or end bud. The continued uneven growth causes the tail bud to extend and curl beneath the hind gut. During the fifth and sixth weeks, the trunk ends in a conspicuous tail containing 10–12 caudal vertebrae. The distal portion lacks bone and is composed of mesodermal elements. During the seventh and eighth weeks, the vertebrated portion retracts into the soft tissue. The nonvertebrated part projects temporarily and then undergoes regression caused by phagocytosis, with the debris-laden macrophages migrating back to the body [11], and it disappears completely at the end of the eighth week. Thus, the presence of human tail can be considered a disturbance in the development of the embryo but not a regression in the evolutionary process. 

Several theories have been developed to explain the development of split cord malformations. Recently, one generally accepted theory suggests that split-cord malformations originate from one basic error occurring around the time when the primitive neuroenteric canal closes. The basic error is the formation of an accessory neuroenteric canal between the yolk sac and amnion, which is subsequently invested with mesenchyme to form an endomesenchymal tract that splits the notocord and neural plate [12, 13]. Pluripotential cells of the endomesenchymal tract could develop into a variety of tissues consisting principally of mesodermal elements [12, 13]. Although the concurrence of a human tail and split cord malformations maybe a mere coincidence, it is tempting to assign a common embryological origin to both events. As already described, during the fetal development, the presence of human tail can be considered a disturbance in the development of the embryo process. Thus, we inferred that disorders of secondary neurulation and abnormal regression of the embryonic tail bud may be the principal cause of this condition, but the exact mechanism is not known. Further investigation into the cellular and molecular biology of normal human spinal cord development may elucidate these variations more clearly.

With the inspiration from previous reports and recent advances of image technology, we consider that the caudal appendage called a true tail is better thought of as being a benign condition, a prolongation beyond the coccygeal or midgluteal region, and should not be associated with any underlying malformation [3, 14, 15]. Simple excision is enough. In contrast, like other lumbosacral skin lesions, the caudal appendages mostly occurring with spina bifida occulta or spinal dysraphism are pseudotails. The appendage is only a cutaneous marker of underlying spinal dysraphism since the skin and nerve systems are related by their similar ectodermal origin. Surgical excision is not done only for cosmetic reasons. Further preoperative examination and complex surgical intervention are usually necessary. 

In conclusion, the caudale appendage is not a medical difficulty to treat. But before the choice of the manner of treatment, it is necessary to evaluate the patient carefully in case of coexisting with the lesions of intraspinal component. After the operation, long-term followup for tethered cord in the patient is necessary.
